# Preventive Effects of Probiotic and Postbiotic *Lacticaseibacillus paracasei* HY2782 on DSS-induced Colitis in Mice: Comparable Efficacy of Live and Heat-Killed Forms

**DOI:** 10.4014/jmb.2512.12027

**Published:** 2026-01-21

**Authors:** Daehyeop Lee, Hyeonjun Gwon, Ji-Woong Jeong, Joo-Yun Kim, Jae-Jung Shim, Jae-Hwan Lee

**Affiliations:** R&BD Center, hy Co., Ltd., Yongin-si 17086, Republic of Korea

**Keywords:** Probiotics, Heat-killed probiotics, Postbiotics, Inflammatory bowel disease, Ulcerative colitis

## Abstract

Ulcerative colitis is chronic inflammatory bowel disease characterized by intestinal inflammation and barrier dysfunction. Probiotics and postbiotics have been proposed as dietary interventions for intestinal health; however, their comparative preventive effects remain unclear. In this study, we evaluated the preventive effects of probiotic and postbiotic forms of *Lacticaseibacillus paracasei* HY2782 in a dextran sulfate sodium (DSS)-induced colitis mouse model. Male C57BL/6 mice were orally administered live or heat-killed HY2782 prior to DSS exposure. Disease activity index, colon length, histopathological damage, inflammatory cytokine expression, and intestinal barrier-related gene expression were assessed, and gut microbiota composition was analyzed using 16S rRNA gene sequencing. Both probiotic and postbiotic forms of HY2782 significantly alleviated DSS-induced colitis, as evidenced by reduced disease activity, preserved colon length, improved histological features, and suppressed expression of pro-inflammatory cytokines. In addition, HY2782 treatment restored the expression of tight junction-related genes in colonic tissue. Gut microbiota analysis revealed limited but specific compositional changes following HY2782 administration, with no marked differences between live and heat-killed forms. These findings demonstrate that both probiotic and postbiotic forms of HY2782 exert preventive effects against DSS-induced colitis with no statistically significant differences between the two preparations. This suggests that HY2782 has significant potential as a versatile functional ingredient in both live and inactivated forms for the prevention of inflammatory bowel diseases, although further studies are needed to fully elucidate their distinct mechanistic roles.

## Introduction

Inflammatory bowel disease (IBD), which includes Crohn’s disease (CD) and ulcerative colitis (UC), is a group of chronic gastrointestinal disorders characterized by inflammation, chronic diarrhea, abdominal pain, and rectal bleeding [1-3]. The incidence and prevalence of UC are rising globally; its precise pathogenesis remains incompletely defined [4]. Increasing evidence suggests that UC develops through a multifactorial interplay involving genetic susceptibility, environmental exposures, microbial infections, immune dysregulation, and gut microbiota alterations [5]. Dysbiosis, or disruption of the intestinal microbial community, is particularly implicated in UC onset, as it impairs the epithelial barrier, promotes mucosal immune activation, and drives pro-inflammatory cytokine production. This compromise in epithelial integrity increases intestinal permeability, accelerating the progression of the disease. In this context, tight junction proteins (TJPs), which are essential for maintaining intestinal epithelial barrier function, play a key role in UC pathophysiology [6]. Collectively, these interrelated factors support the view that UC is an immune-mediated inflammatory disorder driven by disturbances within the intestinal ecosystem [7].

Current therapeutic agents, including corticosteroids, 5-aminosalicylic acid (5-ACA) derivatives, immunomodulators, and biologics, aim to reduce inflammation for managing IBD. However, these treatments are frequently associated with notable adverse effects, ranging from nephrotoxicity and drug hypersensitivity to gastrointestinal symptoms such as abdominal pain, nausea, vomiting, and exacerbation of diarrhea [8, 9]. Moreover, several therapeutic challenges remain, including primary unresponsiveness or secondary loss of response to treatment (*e.g.*, patients ceasing to respond after an initial benefit) and inability to address the underlying microbiota imbalance [10]. These limitations have redirected attention toward interventions, particularly those capable of modulating mucosal barrier integrity and restoring gut microbiota homeostasis. Within this therapeutic framework, probiotics and postbiotics have emerged as potential modalities to reinforce epithelial barrier function, regulate mucosal immune activity, and restore microbial balance.

Probiotics, defined as live microorganisms that confer health benefits to the host, modulate the immune system and enhance the epithelial barrier function through microbial signaling, metabolite production, and host-microbe interaction [11, 12]. Postbiotics, by contrast, are non-viable microbial cells or components derived from probiotics, exerting similar beneficial effects, including immunomodulation and mucosal barrier reinforcement [13, 14]. Given the pathological hallmarks of UC, including mucosal inflammation, impaired barrier integrity, and dysbiosis, both probiotics and postbiotics represent distinctive therapeutic approaches. Probiotics achieve their effects through viability-associated functions like colonization, metabolic activity, and direct immune modulation, while postbiotics provide increased stability and safety by acting independently of microbial viability [15-17]. The therapeutic efficacy of these two forms may vary based on the disease context, with probiotics offering sustained effects through microbial ecosystem modulation, and postbiotics providing reproducible, safer outcomes by reducing inflammation and restoring epithelial integrity [18, 19]. Understanding the contribution of probiotics and postbiotics is essential for optimizing UC treatment strategies and exploring optimal personalized therapeutic approaches [20].

In this study, we investigated the preventive potential of *Lacticaseibacillus paracasei* HY2782, in both its probiotic (live) and postbiotic (heat-killed) forms, against DSS-induced colitis. By focusing on the reinforcement of the intestinal epithelial barrier and the modulation of gut microbiota, we sought to elucidate the distinct and shared mechanisms by which these two bacterial formulations mitigate colonic inflammation.

## Materials and Methods

### Preparation of Live and Heat-Killed Probiotics

*L. paracasei* HY2782 (HY2782) was cultured in Man-Rogosa-Sharp broth (Difco Laboratories, USA) at 37°C for 24 h. After incubation, bacterial cells were harvested by centrifugation at 3,000 ×g for 20 min. The supernatants were discarded, and the bacterial pellet was washed twice with sterile phosphate-buffered saline (PBS) and resuspended in PBS to a concentration of 1 × 10^9^ CFU/ml. For heat-killed probiotics preparation, the suspension was subjected to boiling for 20 min in a water bath with gentle mixing. The complete loss of viability was confirmed by observing no visible colonies on MRS agar plates. For heat-killed preparations, bacterial concentrations were expressed as CFU equivalents based on viable counts measured prior to heat treatment. The same bacterial suspensions were subsequently heat-inactivated and used at equivalent cell densities.

### Cell Culture Conditions and Samples Treatment

RAW 264.7 cells and Caco-2 cells were obtained from the American Type Culture Collection (ATCC; USA) and the Korean Cell Line Bank (KCLB; Republic of Korea), respectively. RAW 264.7 cells were cultured in Dulbecco’s Modified Eagle’s Medium (DMEM; Gibco, USA) supplemented with 10% fetal bovine serum (FBS) and 1% penicillin-streptomycin, and the growth medium was changed every 2-3 days. Caco-2 cells were maintained in DMEM containing 10% FBS and 1% antibiotic-antimycotic. Caco-2 cells were differentiated for 21 days, with the culture medium replaced every 2-3 days. All cells were incubated at 37°C in a humidified atmosphere containing 5% CO_2_.

After reaching sufficient confluency, cells were treated with 10^6^ and 10^7^ CFU/ml of live HY2782 (HY2782_6 and HY2782_7, respectively) or with heat-killed HY2782 at equivalent concentrations (HY2782_6K and HY2782_7K, respectively) for 24 h. Subsequently, lipopolysaccharide (LPS, 1 μg/ml) was added, and cells were incubated for an additional 24 h.

### Gene Expression Analysis by qRT-PCR in RAW 264.7 and Caco-2 Cells

Total RNA was extracted using the Easy-spin Total RNA Extraction Kit (iNtRON Biotechnology, Republic of Korea). Complementary DNA (cDNA) was synthesized from 2 μg of total RNA using the Omniscript Reverse Transcription Kit (Qiagen, Germany). qRT-PCR was conducted using TaqMan Gene Expression Assays (Applied Biosystems, USA).

For RAW 264.7 cells, the following targets were analyzed: tumor necrosis factor (*TNF-α*, Mm00443258_m1), interleukin 1 beta (*IL-1β*, Mm00434228_m1), interleukin 6 (*IL-6*, Mm00446190_m1), nuclear factor of kappa B (*NF-κB*, Mm00476361_m1), and toll-like receptor 4 (*TLR4*, Mm00445273_m1). For Caco-2 cells, the target genes included tight junction protein 1 (*TJP1*, Hs01551871_m1), occludin (*OCLN*, Hs05465837_g1), claudin 1 (*CLDN*, Hs00221623_m1), *TNF-α* (Hs00174128_m1), and *IL-1β* (Hs01555410_m1).

Relative mRNA expression levels were calculated using the 2^-ΔΔCT^ method. Gene expression in RAW 264.7 cells was normalized to *GAPDH* (Mm99999915_g1), and expression in Caco-2 cells was normalized to *GAPDH* (Hs02786624_g1) expression.

### Animals Study Design

Male C57BL/6 mice (6 weeks old) were purchased from Dooyeol Biotech (Republic of Korea) and acclimated for one week prior to experiments. The mice were housed under conditions at a temperature of 20-22°C with 40-60% humidity, a 12 h light/dark cycle, and free access to standard rodent diet and water. All animal studies were approved by the Institutional Animal Care and Use Committee (IACUC) of hy Co. Ltd. (approval number, AEC-2025-0002-Y).

After acclimation, the mice were randomly assigned into five groups (*n* = 8 per group): CON, normal group; DSS, 2% DSS-induced colitis group; SULF, 2% DSS + sulfasalazine (100 mg/kg/day, positive control); HY2782L, 2% DSS + live HY2782 probiotic (1 × 10^9^ CFU/ml); HY2782K, 2% DSS + heat-killed HY2782 postbiotic (1 × 10^9^ CFU/ml). Mice were orally administered sulfasalazine or each sample at a fixed volume of 200 μl daily. The CON and DSS groups administered an equivalent volume of PBS for the same duration.

Mice were orally administered HY2782 for a total of 12 days. Mice were first administered samples for 7 days. After this pre-treatment period, drinking water in all groups except the CON group was replaced with 2% DSS (MP Bio-medicals, USA; molecular weight 36,000-50,000 Da) for 5 days to induce colitis. Throughout both the pre-treatment and DSS induction periods, mice continued to be orally administered sulfasalazine or both forms of HY2782, followed by 2 days for recovery.

During the experimental period, body weight, food intake, and water consumption were recorded daily. On the final day of the experiment, mice were euthanized using CO_2_ gas, and blood, colon, and cecum tissues were collected for further analyses.

### Determination of Disease Activity Index (DAI) Scores

The DAI was evaluated based on the mean of three parameters: body weight loss, stool consistency, and hematochezia. Body weight loss was scored on a scale of 0-4 as follows: 0, no change; 1, 0-5% loss; 2, 5-10% loss; 3, 10-15% loss; and 4, >15% loss. Stool consistency was scored from 0 to 3, where 0 indicated normal stool, 2 indicated loose or soft stool, and 3 indicated watery diarrhea. The degree of hematochezia was scored from 0 to 4, with 0 representing no detectable blood, 2 indicating the presence of occult or visible traces of blood in the stool, and 4 indicating gross rectal bleeding visually observed.

### Histological Analysis Using Hematoxylin and Eosin (H&E) Staining

Colon tissues were fixed in 10% formalin (Sigma Aldrich, USA) at room temperature for 24 h. After that, the tissues were embedded in paraffin and sectioned. The paraffin sections were stained with hematoxylin and eosin (H&E). The images were obtained using a Zeiss Axiovert 100 M microscope (Carl Zeiss AG, USA). Histological damage was evaluated using a composite scoring system. Inflammatory cell infiltration, crypt damage, and mucosal erosion were each scored individually on a scale of 0 to 3 according to severity, and the individual scores were summed to obtain a total histological score for each sample (DooYeol Biotech).

### Gene Expression Analysis by Quantitative Real-Time Polymerase Chain Reaction (qRT-PCR) in Colon Tissues

Total RNA from distal colon tissues was extracted, and cDNA was synthesized as mentioned above. The target genes analyzed in this study were as follows: *TJP1* (Mm01320638_m1), *TJP2* (Mm00495620_m1), occludin (Mm00500910_m1), *IL-1β* (Mm00434228_m1), and *TNF-α* (Mm00443258_m1). The relative mRNA expression levels were calculated using the 2^-ΔΔCT^ method, and all data were normalized to *GAPDH* (Mm99999915_g1) expression.

### Measurement of Pro-Inflammatory Cytokines in Serum

Blood samples were centrifuged at 3,000 ×g for 20 min at 4°C to obtain serum. The concentration of pro-inflammatory cytokines, including TNF-α, IL-1β, IL-6, and KC, was measured using the Mouse High Sensitivity T Cell Magnetic Bead Panel (Merck Millipore, USA). The cytokine measurements were performed using the Luminex Multiplex Assay system (Thermo Fisher Scientific, USA) at LABISKOMA (Republic of Korea).

### DNA Extraction and 16S rRNA Gene Sequencing

Total DNA was extracted from the cecum, and the composition of the microbiota was analyzed through 16S rDNA sequencing using a next-generation sequencing (NGS) platform (Illumina, USA). The universal primer sequences employed for sequencing were as follows: V3-F (5’-TCGTCGGCAGCGTCAGATGTGTATAAGAGACAGCCTACG GGNGGCWGCAG-3') and V4-R (5'-CTCGTGGGCTCGGAGATGTGT ATAAGAGACAGG ACTACHVGGTATCTAATCC-3'). DNA extraction and 16S rDNA sequencing were conducted at Macrogen (Republic of Korea) using an Illumina MiSeq i100 Plus instrument (Illumina).

### Analysis of 16S rRNA Gene Sequencing Data

Amplicon sequencing data were processed with QIIME2 (version 2023.9). Raw sequences were first demultiplexed using the q2-demux plugin, followed by filtering and denoising of low-quality sequences with the DAD2 plugin to generate amplicon sequence variants (ASVs). The ASVs were aligned using MAFFT, and a rooted phylogenetic tree was constructed with FastTree 2 for subsequent phylogenetic analysis. Taxonomic assignment was carried out with the q2-feature-classifier plugin, utilizing the pre-trained SILVA 138 reference database (99% identity threshold). Alpha diversity metrics were computed to assess within-sample diversity using Faith’s Phylogenetic Diversity (Faith’s PD). The Kruskal-Wallis test was employed to evaluate group differences in alpha diversity. Beta diversity was measured weighted UniFrac distance matrices, with results visualized via Principal Coordinates Analysis (PCoA). Group Differences in microbial community composition were analyzed using Permutational Multivariate Analysis of Variance (PERMANOVA). Taxonomic composition was assessed by comparing the relative abundances of microbial taxa across groups. Taxa with a relative abundance of ≥ 1% in at least one group were categorized as major taxa, while others were grouped as minor taxa. Differentially abundant taxa were identified using LEfSe (Linear Discriminant Analysis Effect Size) with a significance threshold of LDA score > 2.0. Spearman’s rank correlation coefficient was used to examine correlations between gut microbiota relative abundance and biochemical markers, with analyses performed in R (Version 3.6.6). All sequencing data are available in the NCBI Sequence Read Archive under the accession number PRJNA1308642.

### Statistical Analysis

All data were expressed as mean ± standard deviation (SD). Differences among multiple groups were analyzed using one-way analysis of variance (ANOVA), followed by Tukey’s post hoc test. Statistical analyses were performed using GraphPad Prism version 6 (GraphPad Software, USA), and *p* < 0.05 was considered statistically significant.

## Results

Before evaluating the *in vivo* efficacy, the anti-inflammatory potential of HY2782 was confirmed in RAW 264.7 macrophages (Fig. S1). Subsequently, we assessed whether these protective effects extended to the intestinal epithelial barrier using Caco-2 cells.

### Effects of Live and Heat-Killed HY2782 on Gene Expression in Caco-2 Cells

**Fig. 1:**
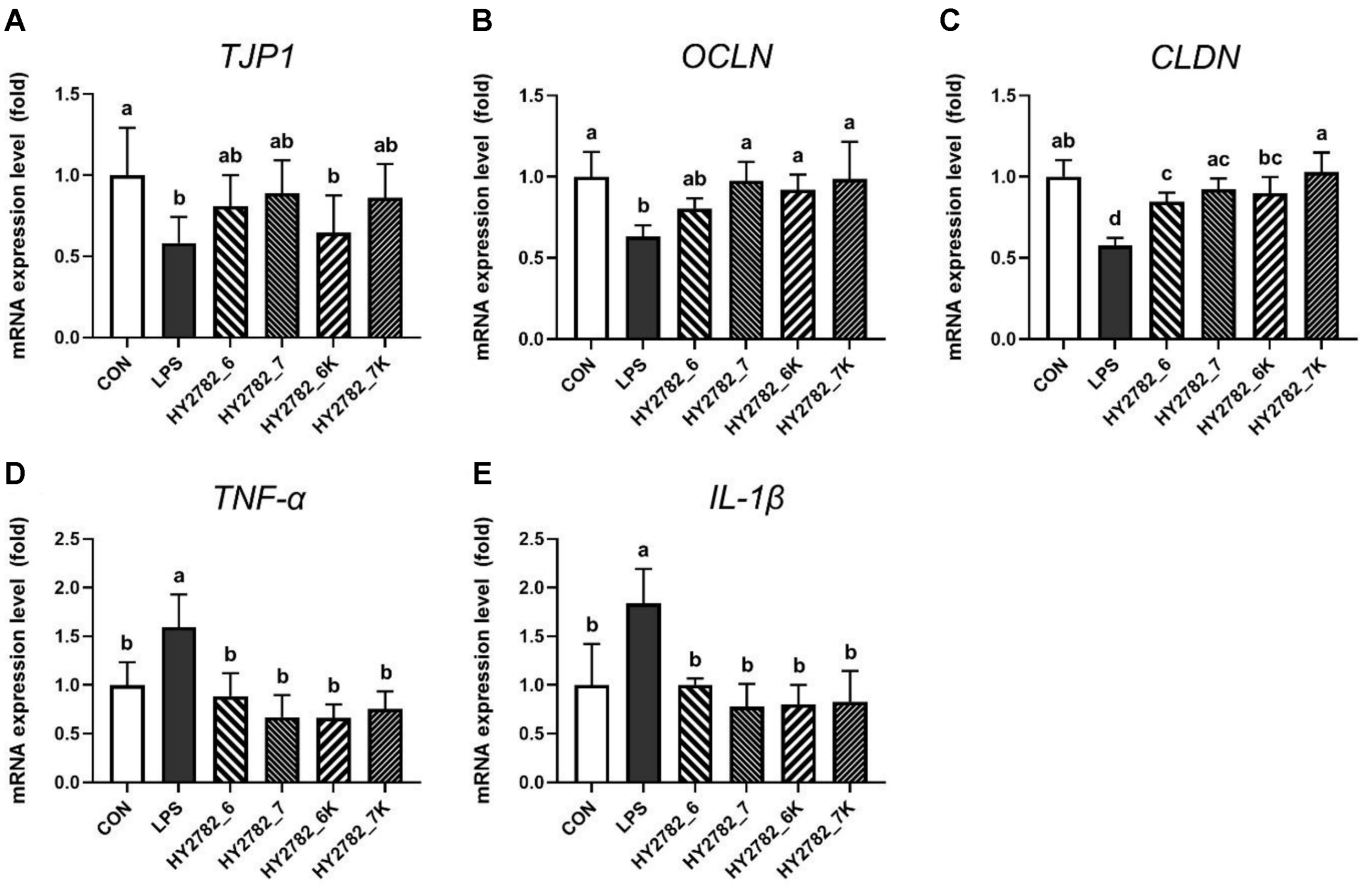
Effects of live and heat-killed HY2782 on gene expression in LPS-induced Caco-2 cell model. The mRNA expression levels of (**A**) tight junction protein 1 (*TJP1*), (**B**) occludin (*OCLN*), (**C**) claudin 1 (*CLDN*), (**D**) *TNF-α*, and (**E**) *IL-1β* were analyzed by quantitative real-time PCR. Data are presented mean ± SD. Different letters indicate significant differences (*p* < 0.05).

The effects of live and heat-killed HY2782 on tight junction and inflammation-related genes were evaluated in the LPS-induced Caco-2 cell model. Treatment with LPS significantly decreased the expression of tight junction genes, *TJP1*, *OCLN*, and *CLDN*, by 0.58-, 0.63-, and 0.58-fold, respectively, compared to the control. The expression of these three tight junction genes was dose-dependently restored following treatment with both live and heat-killed HY2782. Specifically, the expression of the *TJP1* gene was significantly restored in the HY2782_6 group (0.81-fold) and the HY2782_7 group (0.89-fold). Treatment with two different doses of heat-killed HY2782 also upregulated *TJP1* expression, however, there was no significant difference in the HY2782_6K group. The LPS-induced downregulation of the *OCLN* gene was significantly restored following treatment with both live and heat-killed HY2782. Low-dose and high-dose treatments with live HY2782 resulted in a 0.80- and 0.97-fold increase in expression, respectively, while treatment with heat-killed HY2782 led to a 0.92- and 0.99-fold mRNA expression. Similarly, the expression of *CLDN* was also upregulated, with significant increases observed in the HY2782_6 group (0.85-fold), HY2782_7 group (0.92-fold), HY2782_6K group (0.90-fold), and HY2782_7K group (1.03-fold) (Fig. 1A-1C).

In addition, the effects of live and heat-killed HY2782 on the expression of pro-inflammatory cytokines *TNF-α* and *IL-1β* were evaluated in the LPS-induced Caco-2 cell model. Treatment with LPS significantly increased the expression of both TNF-α and IL-1β to 1.59- and 1.84-fold of the control, respectively. However, treatment with both live and heat-killed HY2782 led to restoration of expression across all groups. In the HY2782_6 group, *TNF-α* expression was restored to 0.88-fold, and *IL-1β* expression was restored to 1.00-fold, nearly returning to the control levels. The HY2782_7 group showed a more modest recovery, with *TNF-α* expression restored to 0.67-fold and *IL-1β* expression restored to 0.78-fold. Likewise, treatment with heat-killed HY2782 resulted in restoration of gene expression. In the HY2782_6K group, *TNF-α* and *IL-1β* expression were restored to 0.66-fold and 0.80-fold, respectively. And the expression of *TNF-α* and *IL-1β* was significantly downregulated to 0.75- and 0.83-fold, respectively, in the HY2782_7K group (Fig. 1D and 1E).

### Effects of Live and Heat-Killed HY2782 on Physiological Parameters

**Fig. 2:**
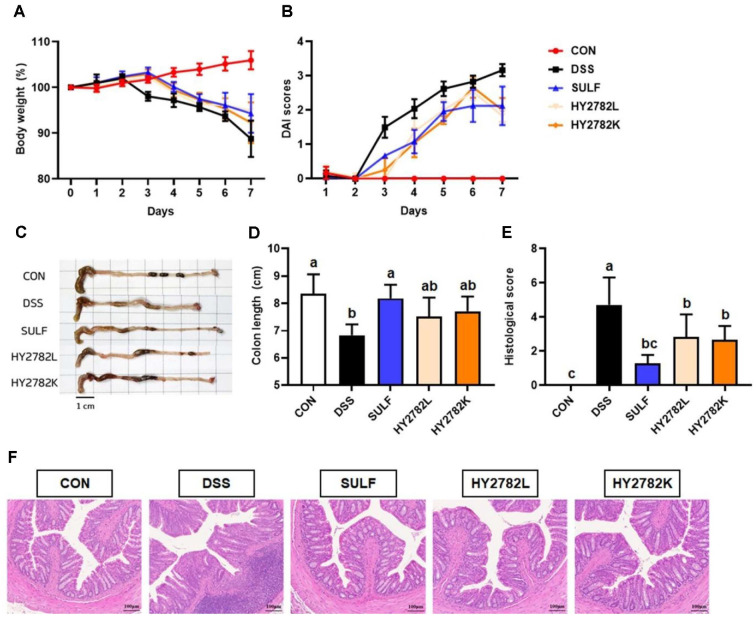
Effects of live and heat-killed HY2782 on physiological and histological parameters in DSS-induced colitis mouse. (**A**) Body weight change (%), (**B**) DAI score, (**C**) colon tissue morphology, (**D**) colon length, (**E**) histological score, and (**F**) representative images of histological changes. Histological scores represent total composite scores obtained by summing individual parameters (each scored 0–3), including inflammatory cell infiltration, crypt damage, and mucosal erosion. Data are presented mean ± SD. Different letters indicate significant differences (*p* < 0.05).

The effects of the live or heat-killed HY2782 on body weight and DAI score are evaluated. When the initial body weight was set to 100%, the CON group exhibited a normal increase throughout the experimental period, reaching 105.92 ± 2.01%. In contrast, the DSS group showed a pronounced reduction in body weight, decreasing to 88.78 ± 3.97%. Treatment with sulfasalazine (SULF group) mitigated this weight loss, resulting in a final weight of 94.29 ± 4.22%. The HY2782L and HY2782K groups similarly alleviated DSS-induced weight loss, maintaining body weights of 94.89 ± 2.78% and 92.26 ± 4.45%, respectively (Fig. 2A).

DAI scores were determined based on body weight change, stool consistency, and fecal blood. The CON group showed no alterations in these parameters and maintained a DAI score of 0 throughout the study. In the DSS group, the DAI score began to increase on day 3 (1.50 ± 0.31) and continued to rise, reaching 3.17 ± 0.18 on day 7. In contrast, the SULF group showed a milder progression, with the score increasing to 0.67 on day 3 and 2.13 ± 0.56 on day 7. The HY2782L and HY2782K groups also demonstrated improvement relative to the DSS group, with DAI scores beginning to rise on days 2 and 3, respectively, and reaching 1.83 ± 0.18 and 2.00 ± 0.36 by day 7 (Fig. 2B).

### Effects of Live and Heat-Killed HY2782 on Histological Parameters

In the DSS-induced colitis mouse model, administration of HY2782 probiotics and postbiotics improved physiological indicators, including colon length and histological scores. DSS treatment markedly shortened colon length. The CON group exhibited a normal colon length of 8.36 ± 0.71 cm, whereas DSS exposure significantly reduced the length to 6.83 ± 0.41 cm. Treatment with sulfasalazine alleviated this reduction, restoring colon length to 8.19 ± 0.50 cm. Similarly, the HY2782L and HY2782K groups showed significant recovery, with colon length of 7.51 ± 0.70 cm and 7.71 ± 0.54 cm, respectively (Fig. 2C and 2D).

Histological evaluation of colon tissues was performed using H&E staining to assess morphological changes. The total histological score was determined by summing the individual scores for inflammatory cell infiltration, crypt damage, and mucosal erosion. The DSS group exhibited marked inflammatory cell infiltration, crypt damage, and signs of edema. The histological score in the DSS group was significantly elevated to 4.71 ± 1.60 compared with the CON group. In contrast, the SULF group demonstrated substantial improvement, with a reduced score of 1.29 ± 0.49. Both HY2782L and HY2782K groups exhibited the restored histological levels, resulting in scores of 2.83 ± 1.33 and 2.67 ± 0.82, respectively, indicating improved colon structure and reduced inflammation and crypt injury (Fig. 2E and 2F).

### Effects of Live and Heat-Killed HY2782 on mRNA Expressions of Genes Related to Tight Junctions and Inflammation

**Fig. 3:**
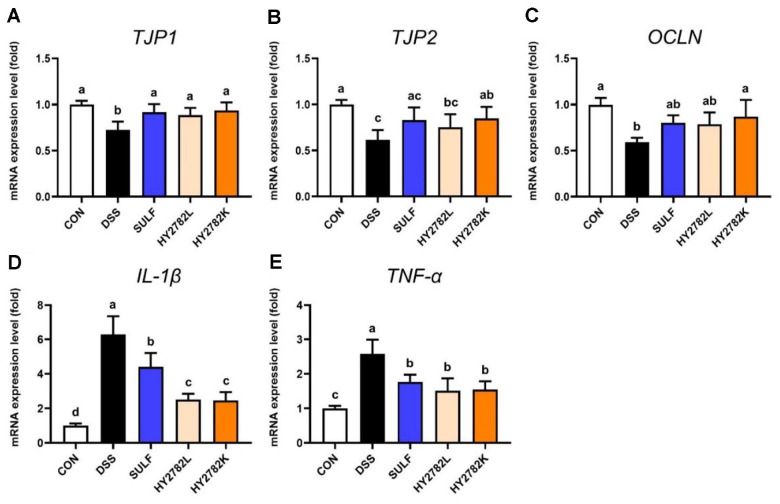
Effects of live and heat-killed HY2782 on gene expression in the colon tissue of DSS-induced colitis mouse. The mRNA expression levels of (**A**) *TJP1*, (**B**) *TJP2*, (**C**) *OCLN*, (**D**) *IL-1β*, and (**E**) *TNF-α* were analyzed by quantitative real-time PCR. The results are presented as the mean ± SD. Different letters indicate significant differences (*p* < 0.05).

The effects of HY2782 probiotics and postbiotics on the expression of tight junction and inflammation-related genes in colon tissue were evaluated. DSS treatment markedly reduced the mRNA expression levels of the tight junction genes, *TJP1*, *TJP2*, and *OCLN* to 0.73 ± 0.09, 0.63 ± 0.11, and 0.61 ± 0.05 relative to the CON group, respectively. In the SULF group, the gene expression levels were restored to 0.92 ± 0.09, 0.85 ± 0.14, and 0.82 ± 0.09. Likewise, both the HY2782L and HY2782K groups significantly recovered the expression levels of all three tight junction genes (Fig. 3A-3C).

Then, the expression of the pro-inflammatory cytokines *IL-1β* and *TNF-α* was examined. DSS treatment led to a significant increase in their expression, reaching 6.29 ± 1.07 and 2.58 ± 0.41 relative to the CON group. In contrast, administration of sulfasalazine reduced their expression to 4.41 ± 0.08 and 1.77 ± 0.21. Administration of HY2782 probiotics further decreased *IL-1β* and *TNF-α* expression to 2.50 ± 0.35 and 1.51 ± 0.36, respectively, while the HY2782 postbiotics showed a similar reduction, 2.46 ± 0.49 and 1.54 ± 0.24, respectively. These results indicate that both live and heat-killed HY2782 effectively attenuate DSS-induced inflammatory responses and preserve tight junction integrity in colon tissue (Fig. 3D and 3E).

### Effect of Live and Heat-Killed HY2782 on Inflammatory Parameters in Serum

**Fig. 4:**
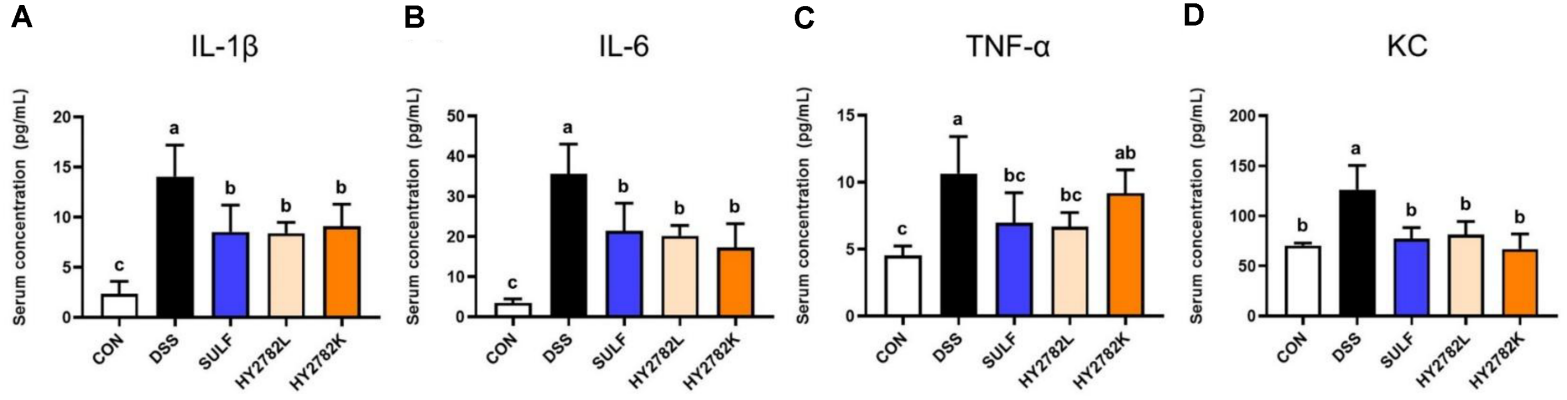
Effects of live and heat-killed HY2782 on the concentration of inflammatory parameters in serum. Concentration of (**A**) *IL-1β*, (**B**) *IL-6*, (**C**) *TNF-α*, and (**D**) KC were analyzed. The results are presented as the mean ± SD. Different letters indicate significant differences (*p* < 0.05).

The serum concentration of the pro-inflammatory cytokines IL-1β, IL-6, and TNF-α, as well as chemokine KC, was analyzed. In the CON group, IL-1β, IL-6, and TNF-α levels were 2.35 ± 1.26 pg/ml, 3.49 ± 0.97 pg/ml, and 4.51 ± 0.73 pg/ml, respectively. After DSS administration, their levels significantly elevated to 14.06 ± 3.13 pg/ml, 35.54 ± 7.44 pg/ml, and 10.65 ± 2.76 pg/ml, respectively. Supplementation with sulfasalazine markedly reduced these cytokines 8.53 ± 2.67 pg/ml, 21.40 ± 6.88 pg/ml, and 6.95 ± 2.26 pg/ml. Similarly, the HY2782L group showed significant reductions, with levels of 8.40 ± 1.07 pg/ml (IL-1β), 20.14 ± 2.59 pg/ml (IL-6), and 6.47 ± 1.05 pg/ml (TNF-α). The HY2782K group also attenuated cytokine production, with levels of 9.10 ± 2.19 pg/ml, 17.25 ± 5.93 pg/ml, and 9.20 ± 1.73 pg/ml, respectively (Fig. 4A-4C).

Similarly, chemokine KC levels in serum were 70.26 ± 2.60 pg/ml in the CON group but were significantly increased to 126.13 ± 24.33 pg/ml following DSS exposure. In the SULF group, the KC concentration was reduced to 77.20 ± 11.07 pg/ml. The HY2782L and HY2782K group also significantly reduced KC levels, reaching 81.04 ± 13.46 pg/ml and 66.92 ± 15.04 pg/ml, respectively (Fig. 4D).

### Effects of Live and Heat-Killed HY2782 on the Composition of the Gut Microbiota

**Fig. 5:**
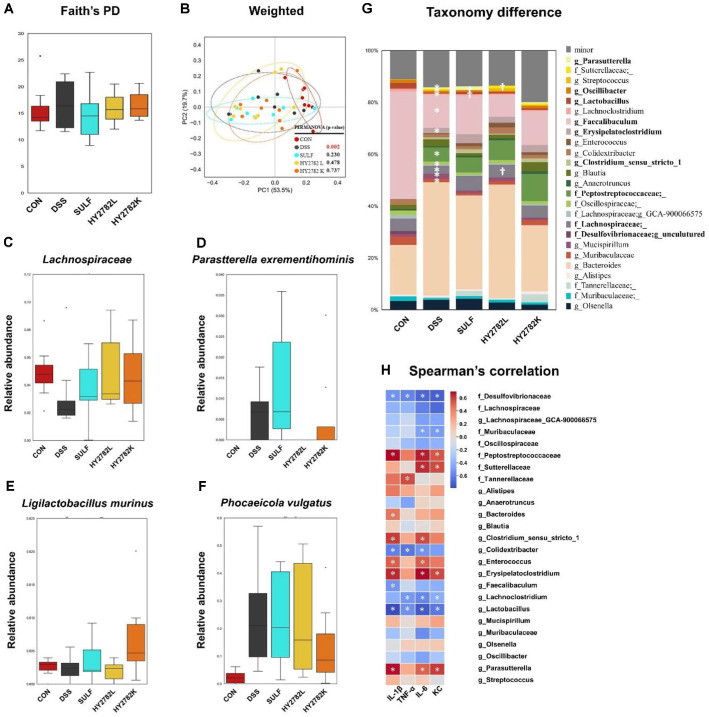
Effects of live and heat-killed HY2782 on gut microbiota diversity and composition in a DSS-induced colitis mouse model. (**A**) Faith’s phylogenetic diversity (PD) and (**B**) weighted UniFrac distances. The species-level abundance of (**C**) *Lachnospiraceae*, (**D**) *Parasutterella excrementihominis*, (**E**) *Ligilactobacillus murinus*, and (**F**) *Phocaeicola vulgatus* and (**G**) relative abundance of bacterial genera. (**H**) Spearman’s correlation between changes of gut microbiota and serum level inflammatory parameters. Comparison between the CON and DSS groups (*, LDA scores > 2.0) and between the DSS and treatment groups (†, LDA scores > 2.0).

Alpha diversity was assessed using Faith’s PD. Faith’s PD showed a tendency to increase following DSS treatment. In contrast, supplementation with sulfasalazine, live or heat-killed HY2782, did not result in significant changes to alpha diversity. Beta diversity analysis revealed significant shifts in microbial community composition after DSS induction, whereas minimal changes were observed in the HY2782L and HY2782K groups (Fig. 5A and 5B).

At the genus level, the DSS group exhibited an increased abundance of *Peptostreptococcaceae*, *Erysipelatoclostridium*, *Parasutterella*, and *Enterococcus*, while showing a reduction in *Bifidobacterium*, *Lachnoclostridium*, *Lachnospiraceae*, *Lactobacillus*, and *Faecalibaculum*. In contrast, the SULF group showed an increase in *Lactobacillus* and a decrease in *Sutterellaceae*, *Acetatifactor*, and *Akkermansia*. The HY2782L group demonstrated an increase in *Lachnospiraceae* and a decrease in *Parasutterella*. The HY2782K group exhibited increases in *Enterohabdus*, *Peptococcaceae*, and *Tepidibacter*, with a reduction in *Muribaculum* (Fig. 5C-5G).

### Correlation between Inflammatory Markers and Gut Microbial Profiles after Live and Heat-Killed HY2782 Treatment

Correlation analysis revealed strong positive associations of *Peptostreptococcaceae*, *Erysipelatoclostridium*, and *Parasutterella* with IL-1β, TNF-α, IL-6, and KC. In contrast, *Desulfovibrionaceae*, *Bifidobacterium*, *Colidextribacter*, *Lactobacillus*, *Faecalibaculum*, and *Lachnospiraceae* exhibited negative correlations with these inflammatory markers. Notably, *Lactobacillus* showed strong negative correlations with all measured inflammatory indicators (Fig. 5H).

## Discussion

Inflammatory bowel disease (IBD) is a common intestinal disorder affecting people worldwide, yet its underlying mechanisms remain poorly understood [21, 22]. Current treatments often have side effects, emphasizing the need for safer and more effective therapies that can modulate inflammation and restore dysbiosis with fewer complications [23]. Probiotics are beneficial in IBD, aligning with its pathogenesis, as they enhance intestinal health and help manage or prevent intestinal disorders by blocking inflammatory reactions and altering the bacterial flora [24]. However, since probiotics involve the consumption of live bacteria, there are potential limitations associated with their use. The use of heat-killed probiotics, which retain their functional characteristics, including immunomodulatory and barrier-enhancing effects, could minimize such risks or potential side effects [25, 26], while providing comparable antioxidant and anti-inflammatory benefits to their live counterparts [13, 14]. Consequently, we aim to investigate the preventive potential of both live and heat-killed *L. paracasei* HY2782 in alleviating colitis. The primary focus of this study was to understand their distinct mechanisms in reinforcing the intestinal barrier and modulating microbial homeostasis to prevent the onset and progression of colonic inflammation.

In preliminary *in vitro* screening assays, both live and heat-killed HY2782 exhibited anti-inflammatory potential in macrophage-based models, supporting their suitability for subsequent *in vivo* evaluation. Given the limited translational relevance of isolated macrophage responses, the present study focused primarily on epithelial barrier protection and inflammatory outcomes in a DSS-induced colitis mouse model. In this context, inflammatory responses in colitis are known to involve multiple pattern-recognition receptor–associated signaling pathways, including the TLR4–NF-κB axis, which provides a general biological framework for interpreting inflammation-related gene expression changes observed under DSS-induced conditions [27-29].

We also assessed whether these protective effects extended to intestinal epithelial cells, which are crucial for maintaining mucosal barrier integrity in UC. In Caco-2 cells, we examined tight junction-related genes (*TJP1*, *OCLN*, and *CLDN*) and pro-inflammatory cytokines (*TNF-α* and *IL-1β*) to evaluate the effects of live and heat-killed HY2782 on epithelial barrier integrity under inflammatory conditions. Impaired epithelial barrier triggers inflammatory cytokine release, disrupts tight junctions, and increases intestinal permeability, contributing to infection and chronic inflammatory diseases [30]. LPS exposure reduced tight junction gene expression and increased cytokine levels, reflecting epithelial dysfunction. However, treatment with live or heat-killed HY2782 restored tight junction gene expression and suppressed cytokine production, suggesting that both probiotics and postbiotics forms of HY2782 may contribute to the preservation of epithelial barrier integrity under inflammatory conditions.

Based on these *in vitro* findings, which demonstrate that both the probiotic and postbiotic forms of HY2782 can enhance epithelial integrity and modulate inflammatory responses, we next conducted an *in vivo* experiment using a DSS-induced colitis mouse model. The DSS model is widely used for its simplicity, reproducibility, and ability to replicate key features of human UC, including acute epithelial injury, mucosal inflammation, and colon-specific pathology [5, 31]. After DSS induction, DAI scores, calculated based on body weight loss, stool consistency, and fecal bleeding, were significantly increased. Additionally, colon length was markedly reduced, and histological scores, including inflammatory cell infiltration, crypt damage, and ulceration, were elevated compared to the CON group. However, the HY2782L and HY2782K groups demonstrated improvements in these physiological and histological alterations, indicating a reduction in disease severity. While the *in vitro* results indicated that live and heat-killed HY2782 treatments can modulate inflammatory responses and epithelial integrity, *in vivo* findings demonstrated their protective effects against DSS-induced epithelial injury and mucosal inflammation. These results suggest that live and heat-killed HY2782 may modulate colonic inflammation and barrier dysfunction through multiple complementary actions.

UC is characterized by impaired epithelial integrity and disrupted tight junction structures, which compromise the intestinal barrier’s selective permeability, increasing susceptibility to microbial invasion and toxin diffusion [32]. Tight junction proteins such as TJP1, TJP2, and OCLN are essential for maintaining barrier function, so strategies that enhance epithelial integrity could provide an important therapeutic approach for UC [33, 34]. In this study, both HY2782L and HY2782K groups restored the DSS-induced downregulation of *TJP1*, *TJP2*, and *OCLN*, indicating their ability to preserve tight junction integrity under inflammatory conditions. Additionally, the expression of pro-inflammatory cytokines, including *IL-1β* and *TNF-α*, was significantly reduced in the HY2782L and HY2782K groups compared to the DSS group. Balanced cytokine expression is essential for maintaining intestinal homeostasis, whereas IBD is characterized by disrupted cytokine regulation due to excessive pro-inflammatory cytokine production [35]. The suppression of *IL-1β* and *TNF-α* in Caco-2 cells suggests that both HY2782 probiotic and postbiotic can regulate the inflammatory response in DSS-induced colitis, potentially reducing mucosal damage and improving overall disease severity.

Despite these promising findings, some limitations should be acknowledged. While we evaluated serum cytokines at the protein level, our molecular analyses in colonic tissue were limited to mRNA expression, which did not capture post-transcriptional regulation or protein-level alterations in local inflammatory signaling. In addition, cytokine regulation involves complex interactions among various immune cell populations, which were not comprehensively assessed in this study. Although both live and heat-killed HY2782 exhibited protective effects, the exact mechanisms warrant further elucidation. Based on our findings, the comparable efficacy between the two forms likely results from a functional convergence of host-centered responses. In the acute DSS model, which is primarily driven by epithelial injury and innate immune activation, host-mediated pathways such as pattern-recognition receptor (PRR) signaling may play a dominant role in maintaining barrier integrity. Consistent with this, our *in vitro* data showed that both live and heat-killed HY2782 modulated TLR4 and NF-κB expression in macrophages (Fig. S1). This suggests that non-viable components retain the capacity to activate immune pathways that attenuate inflammation and reinforce the intestinal barrier. In this acute injury context, these shared host-mediated mechanisms may override viability-dependent effects, leading to similar phenotypic outcomes. Importantly, this similarity reflects the constraints of an acute injury model rather than identical biological actions. Future studies using chronic or therapeutic models are needed to further distinguish the unique mechanistic pathways of probiotics versus postbiotics.

Furthermore, the serum concentrations of IL-1β, IL-6, TNF-α, and KC were significantly elevated in the DSS group compared to the CON group. These cytokines are key mediators of inflammatory signaling and immune cell activation, playing a pivotal role in exacerbating intestinal inflammation and contributing to tissue damage. Specifically, IL-6 and TNF-α modulate intestinal tight junctions and increase permeability in IBD patients [36, 37]. Excessive IL-1β further increases permeability and activates dendritic cells and macrophages, while KC recruits neutrophils, leading to additional epithelial destruction [38, 39]. There was a significant reduction in the HY2782L and HY2782K groups of these cytokines, suggesting that both forms not only suppressed localized inflammation in the colon but also attenuated the systemic spread of inflammatory responses.

The reduction in serum inflammatory markers and the increased expression of tight junction genes in the HY2782L and HY2782K groups suggest that both probiotics and postbiotics may help reduce inflammation in colitis and improve gut microbiota imbalance [40, 41]. To investigate the relationship between inflammatory markers and microbial alterations, alpha- and beta-diversity analyses were performed. Consistent with previous studies, DSS induction caused a typical dysbiotic shift, characterized by an increase in Faith’s PD [42, 43]. Given that Faith’s PD reflects phylogenetic diversity rather than species richness, its elevation following DSS treatment likely reflects the expansion of phylogenetically diverse opportunistic taxa driven by epithelial barrier disruption and increased intestinal permeability. Although live and heat-killed HY2782 supplementation showed modest trends toward alpha diversity restoration, the severely compromised intestinal environment under acute DSS exposure appeared to limit substantial community-level recovery. Accordingly, beta-diversity analysis revealed marked DSS-driven microbial composition with minimal separation between the supplementation and DSS groups, suggesting that acute inflammatory pressure masked detectable improvements in overall microbial community composition.

After evaluating alpha and beta diversity, taxonomic profiling was performed to assess the microbial groups’ abundance across the CON, DSS, SULF, HY2782L, and HY2782K groups. DSS treatment reduced bacterial families associated with short-chain fatty acids (SCFAs) production, such as *Lachnospiraceae*, *Faecalibaculum*, and *Lactobacillus*, while increasing genera linked to inflammation and epithelial dysfunction, including *Erysipelatoclostridium*, *Peptostreptococcaceae*, and *Parasutterella* [44, 45]. Despite limited recovery in community structure, selective microbial shifts were observed in the HY2782L and HY2782K groups. Live HY2782 treatment increased *Lachnospiraceae* and completely suppressed *Parasutterella excrementihominis*, a bacterium associated with colonic inflammation [46]. In the HY2782K group, inflammation-associated taxa, including *Bacteroides*, were reduced, and *Lachnospiraceae* increased to levels similar to those observed in the HY2782L and SULF groups, suggesting a compositional shift toward microbial taxa commonly associated with intestinal homeostasis. Notably, *Ligilactobacillus murinus*, which supports epithelial integrity, was substantially increased in the HY2782K group, surpassing levels in the CON group, an effect not observed in the HY2782L group. One possible explanation is that differences in bacterial viability may influence host–microbe interactions and microbial competitiveness under DSS-induced inflammatory stress. Viable and non-viable bacterial preparations are known to differ in their interactions with the intestinal environment, which may affect the persistence or expansion of specific taxa during acute inflammation. In the present study, however, the precise mechanisms underlying the differential abundance of specific taxa, including *L. murinus*, were not directly examined and therefore cannot be conclusively determined, although previous studies have suggested that host pattern-recognition receptors such as TLR2 and NOD2 play important roles in mediating epithelial–microbial interactions [47]. In contrast, there was an increase in *Lactobacillus* in the SULF group, suggesting restoration of the colonic barrier, creating a more favorable environment for *Lactobacillus* growth [48]. The SULF group did not show a reduction in *Parasutterella excrementihominis*, which distinguishes its microbial response from that of the HY2782L and HY2782K group. This pattern is consistent with the pharmacological profile of sulfasalazine, which is metabolized in the colon into 5-ACA and primarily exerts anti-inflammatory effects through local immune pathways rather than through modulation of the gut microbiota [49]. Given the limited recovery of overall microbial diversity, the observed taxonomic changes are more appropriately interpreted as being associated with, rather than driving, the anti-inflammatory and barrier-protective effects observed *in vivo*. As a result, sulfasalazine typically induces only limited shifts in microbial composition, explaining why meaningful microbiota restructuring was not observed in this group despite improvements in inflammatory readouts.

*Phocaeicola vulgatus*, also known as *Bacteroides vulgatus*, is commonly found in the colonic microbiota of UC patients, with some exhibiting elevated serum antibodies against its OmpA protein, which promotes bacterial adherence. Protease-producing *P. vulgatus* strains are linked to increased intestinal permeability and active colitis through the secretion of dipeptidyl peptidases [50]. In this study, DSS treatment increased *P. vulgatus* abundance, with a relatively minor reduction in the SULF and HY2782L groups, while a significant decrease in the HY2782K group. Live bacteria likely exert their effects through direct competition with *P. vulgatus* by metabolizing organic acids and creating an unfavorable environment for their growth. In contrast, heat-killed bacteria activate intestinal immune responses, enhancing tight junction protein expression through immune receptors like TLR2 and NOD2, thereby strengthening the epithelial barrier [47]. Additionally, heat-killed bacteria do not induce competitive inhibition as strongly as live bacteria, which aligns with the observed increase in TJP gene expression in the colon in the HY2782K group. The results indicate that live and heat-killed HY2782 suppress *P. vulgatus* through different mechanisms, contributing to improved intestinal barrier function and reduced inflammation in the DSS-induced colitis model. Taken together, the microbiome data indicate that administration of live or heat-killed HY2782 did not induce large-scale restructuring of the gut microbial community under acute DSS-induced inflammatory conditions. Instead, the observed selective taxonomic shifts are more appropriately interpreted as being associated with, rather than driving, the anti-inflammatory and barrier-protective effects observed *in vivo*.

**Fig. 6:**
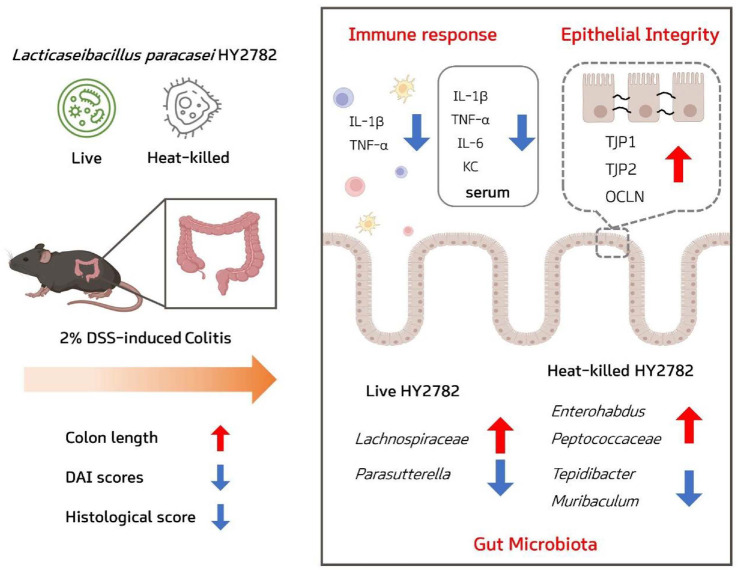
Schematic summary of the preventive effects of live and heat-killed HY2782 on DSS-induced colitis.

In summary, the present findings indicate that *L. paracasei* HY2782, administered as either live or heat-killed formulations, has preventive potential against colitis-associated inflammation and epithelial barrier disruption in a DSS-induced model. In line with this objective, the present study was intentionally designed as a preventive proof-of-concept model, and therapeutic administration following disease onset was not evaluated. Taken together, these findings suggest that both live and heat-killed forms of HY2782 can ameliorate inflammatory responses and epithelial barrier dysfunction primarily through host-centered effects rather than extensive microbiota remodeling (Fig. 6). However, these results should be interpreted with caution, as mechanistic differences between live and inactivated preparations were not fully resolved, and the relatively short duration of administration limits conclusions regarding long-term efficacy and microbiome remodeling. Consequently, additional studies incorporating extended preventive dosing strategies, detailed mechanistic investigations, and diverse colitis models will be required to more definitively establish the preventive potential and translational applicability of HY2782.

## Supplemental Materials

Supplementary data for this paper are available on-line only at http://jmb.or.kr.
