# Supplementary material for: Preventive Effects of Probiotic and Postbiotic Lacticaseibacillus paracasei HY2782 on DSS-induced Colitis in Mice: Comparable Efficacy of Live and Heat-Killed Forms
Source: J Microbiol Biotechnol. 2026 Jan 21;36:e2512027. doi: 10.4014/jmb.2512.12027 (PMC12868948; doi:10.4014/jmb.2512.12027)
Supplement: Supplementary file 1 [file jmb-36-e2512027-supple.pdf]

J. Microbiol. Biotechnol. 2026. 36: e2512027

https://doi.org/10.4014/jmb.2512.12027

Supplementary Figures

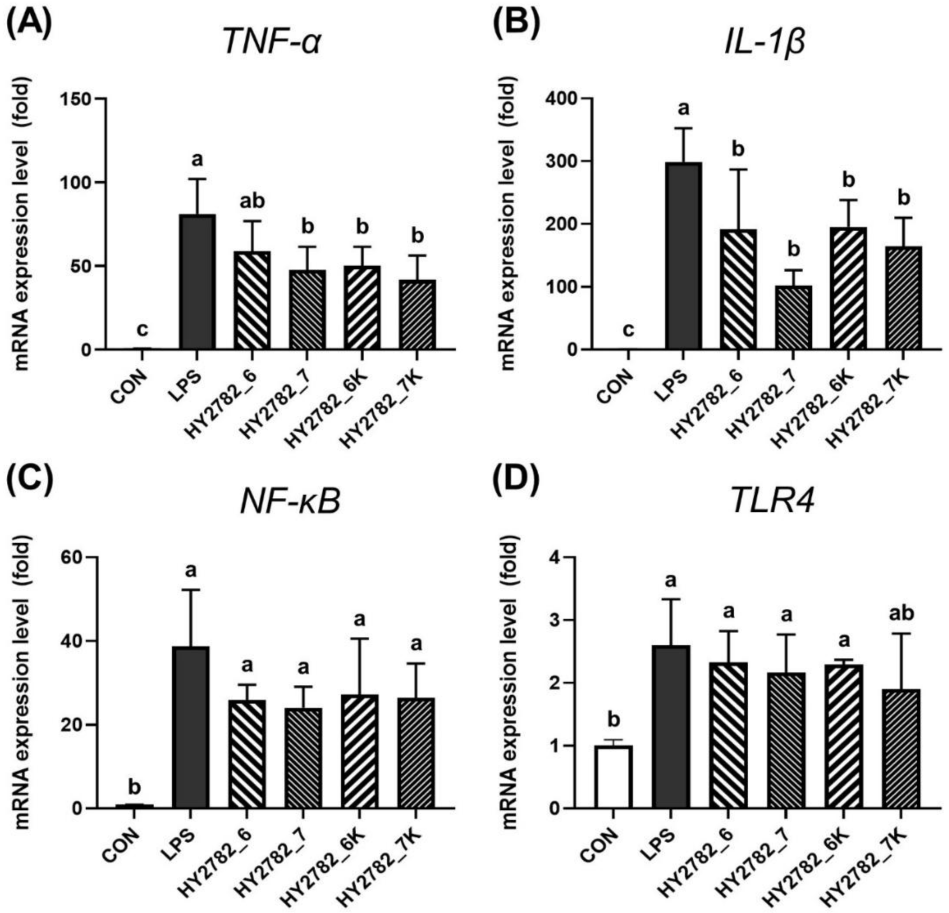

Fig. S1. **Effects of live and heat-killed *Lacticaseibacillus paracasei* HY2782 on gene expression in LPS-induced RAW 264.7 cell model**. The mRNA expression levels of **(A)** tumor necrosis factor (*TNF-α*), **(B)** Interleukin 6 (IL-6), **(C)** interleukin 1 beta (*IL-1β*), **(D)** nuclear factor of kappa B (*NF-κB*), and (E) toll-like receptor 4 (*TLR4*) were analyzed by quantitative real-time PCR. Data are presented mean ± SD. Different letters indicate significant differences ($p < 0.05$).

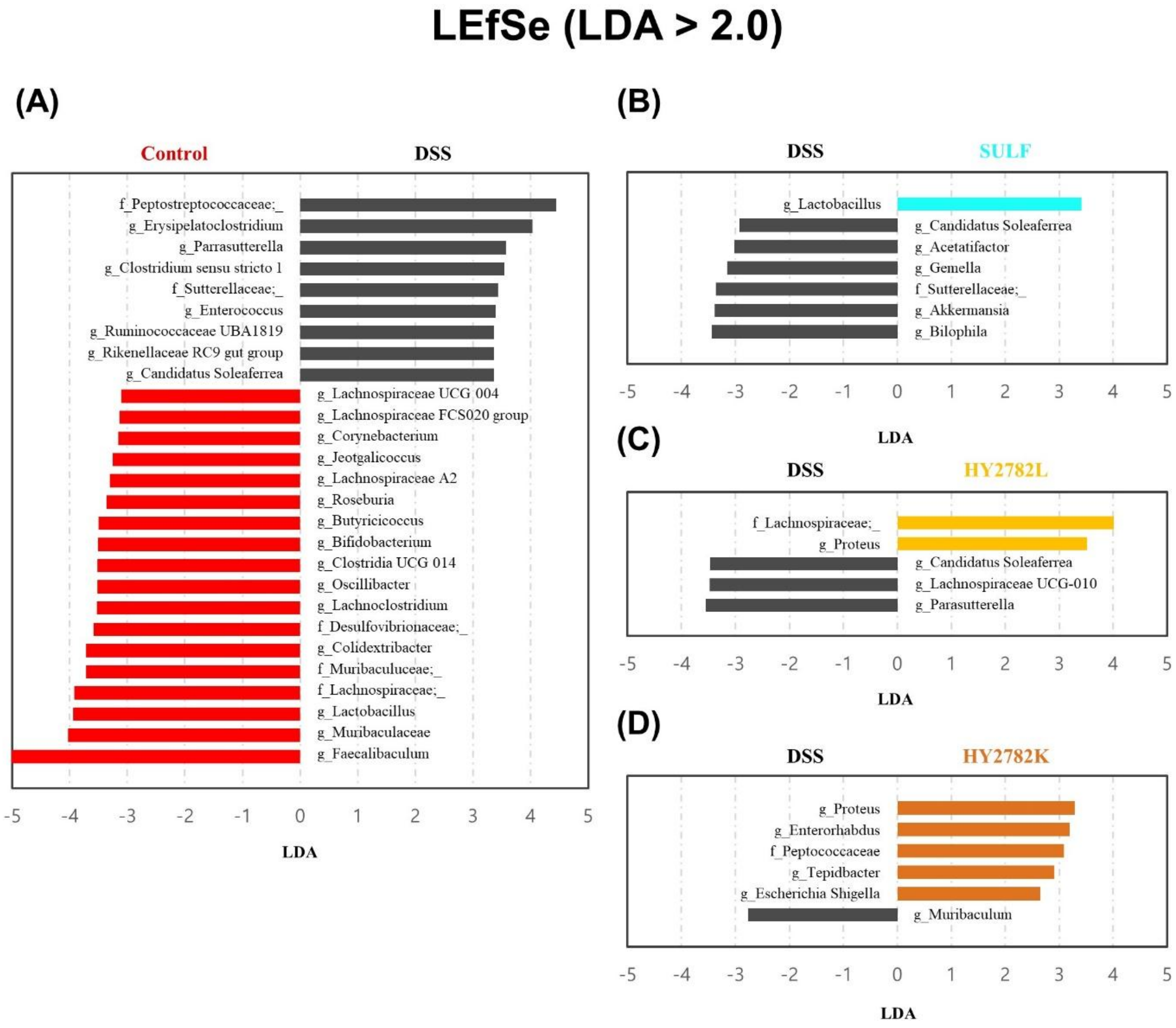

Fig. S2. Taxonomic cladogram of differentially abundant microbial taxa identified by LEfSe analysis (LDA > 2.0 at the genus level) comparing the DSS group with (A) Control, (B) SULF, (C) HY2782L, and (D) HY2782K groups.
